# Dermoid cyst of the pancreas: presentation and management

**DOI:** 10.1186/1477-7819-5-85

**Published:** 2007-08-03

**Authors:** Gianfranco Tucci, Marco Gallinella Muzi, Casimiro Nigro, Federica Cadeddu, Dalia Amabile, Francesca Servadei, Attilio M Farinon

**Affiliations:** 1 Department of Surgery, University Hospital Tor Vergata, Viale Oxford 81 00133 Rome, Italy; 2 Department of Pathology, University Hospital Tor Vergata, Viale Oxford 81 00133 Rome, Italy

## Abstract

**Background:**

Dermoid cyst of the pancreas is a benign, well-differentiated, extremely rare germ cell neoplasm. Published data indicate that differential diagnosis of cystic lesions of the pancreas is challenging and although ultrasonography, computed tomography and magnetic resonance may be useful, radiological findings are often inconclusive and the diagnosis is intraoperative. We report a case of a dermoid cyst of the tail of the pancreas intraoperatively diagnosed and successfully treated with left pancreatectomy. Further, characteristics, preoperative detection and differential diagnosis of this rare pathology are also discussed.

**Case presentation:**

This report documents the findings of a 64-year-old male presenting with a well defined echogenic pancreatic mass on ultrasonography. Computerized Tomography (CT) showed a 5 cm cystic tumor arising from pancreatic tail and Magnetic Resonance Imaging (MRI) suggested a tumor extension to the middle side of the stomach without defined margins. A left pancreatectomy was performed. On surgical specimen, histological evaluation revealed a dermoid cyst of the tail of the pancreas measuring 8.5 × 3.0 cm.

**Conclusion:**

Given the benign nature of the dermoid cyst, surgical resection most likely represents the definitive treatment and cure. In addition, resection is indicated in consideration of the difficulty in diagnosing dermoid cyst preoperatively. However, endoscopic ultrasound and fine needle aspiration cytology have recently been shown to be effective, safe, reliable and cost-saving preoperative diagnostic tools. Therefore, until more cases of dermoid cyst are identified to further elucidate its natural history and improve the reliability of the preoperative diagnostic tools, surgical resection should be considered the standard therapy in order to exclude malignancy.

## Background

Dermoid cyst of the pancreas, also called cystic teratoma, constitutes an unusual entity with only 21 cases, to our knowledge, described in the world literature [1-21]. Mature cystic teratoma of pancreas was first described in 1918 by Kerr [1] and, in 1922, it was included by Primrose in the classification of cystic pancreatic lesions [22].

Teratomas are neoplasms of germ cell origin, able to generate tissues from all the three germ layers (ectoderm, endoderm, and mesoderm) [20,21].

They can be classified as benign, well-differentiated lesions, which are solid or cystic, and solid malignant undifferentiated tumors, named, respectively, mature and immature teratomas on the basis of the presence of immature neuroectodermal elements within the tumour [21].

Mature cystic teratoma is commonly found in the ovary, but may occur in any pathway of ectodermal cell migration, typically in the midline, such as testes, cranium, brain, mediastinum, omentum, retroperitoneum and sacrococcygeal regions. Besides, pancreas is extremely rare as primary site [20].

Clinical presentation of pancreatic dermoid cyst is non specific. Complaints at presentation include abdominal pain, back pain, nausea, vomiting, anorexia, weight loss, fatigue, fever and finally some cases can be diagnosed during a work-up for other diseases [14,20,21].

Dermoid cyst of the pancreas is a true cyst, thus the cyst wall consists of stratified squamous epithelium and underlying connective tissue. At macroscopical evaluation, the cyst contains differentiated tissues from one or more germ cell layers, usually ectodermal; the cyst content appears pasty, "cheesy" or "caseous", with keratinaceous and sebaceous secretions, and it rarely may be clear and serous. Microscopically, the cyst is lined by stratified squamous epithelium and immediately adjacent, dense sub epithelial lymphoid tissue that contains lymphoid follicles and germinal centers.

Differential diagnosis of lesions that mimic cystic neoplasms of the pancreas is still difficult and it is especially hard to differentiate among pancreatic cystic neoplasms. Even though Ultrasonography (US), CT and MRI may be helpful, there are no pathognomonic data for their preoperative recognition.

The diagnostic approach in such patients is debated. Some observers emphasize the difficulties in achieving a diagnosis of certitude without resection, whereas others choose to determine the need for surgery based on the results of Endoscopic Ultrasound (EUS) coupled with US, CT or EUS-guided FNA cytology and histology [23].

Treatment of dermoid cysts consists of surgical removal; clinical observation has not been reported. Surgical procedures previously reported in literature are external drainage in 5 patients, more recently abandoned in consideration of postoperative chronic draining fistula [14], cystectomy in 12; distal pancreatectomy in two (one plus splenectomy) and cystogastrectomy in one case [1-21].

The authors present a case report of a 64-year-old man with a pancreatic dermoid cyst intraoperatively diagnosed; furthermore preoperative detection, differential diagnosis and management of pancreatic dermoid cysts are also discussed.

## Case presentation

**Figure 1:**
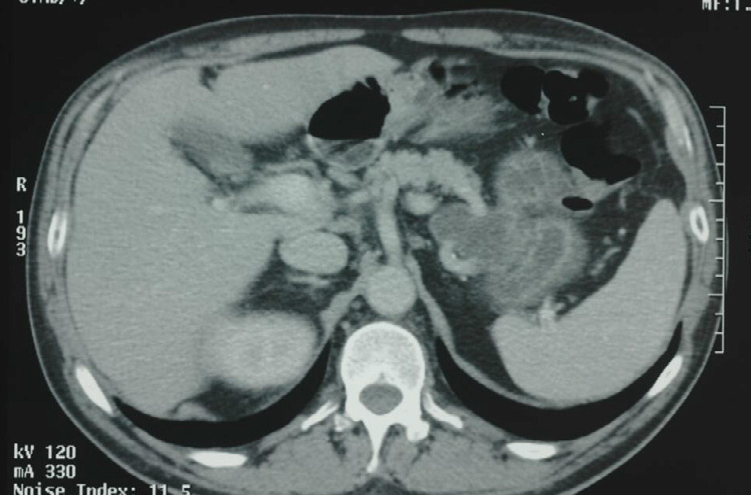
**CT scan of the pancreatic cystic teratoma**. Computed tomography showing a 5 cm cystic fluid mass arising from the tail of the pancreas.

**Figure 2:**
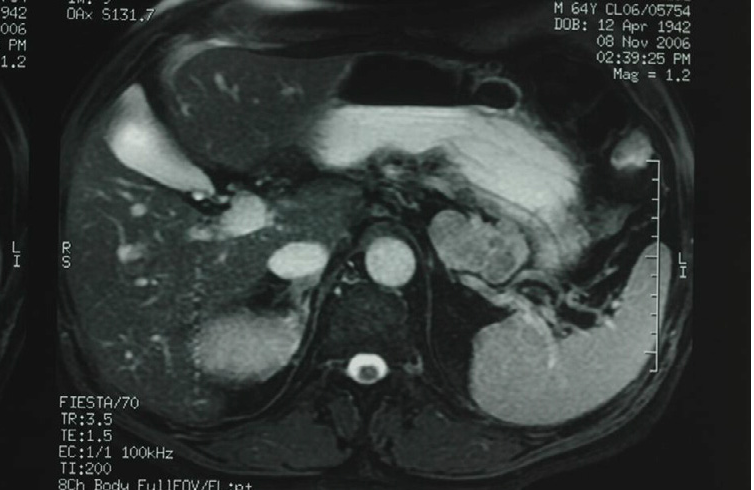
**MRI of the cystic teratoma of the pancreatic tail**. Enhanced magnetic resonance shows a cystic mass arising from the tail of the pancreas extending to the middle side of the stomach without defined margins.

A 64-year-old man, with a history of non-insulin dependent diabetes mellitus, presented with a L4-S1 chronic radiculophaty, in absence of abdominal complaints. Laboratory studies showed normal values except for increase of CA 19-9 levels (CEA, and Ca 19-9 were tested). On imaging investigation, ultrasonography showed a well defined echogenic pancreatic mass, CT scan of abdomen showed a 5 cm low attenuation cystic tumor arising from the pancreatic tail (Figure 1). Although MRI was performed to investigate anatomic relationships and morphologic features of the tumor, the precise origin of the mass couldn't be defined. MRI, in fact, suggested a possible extension of the tumor to the middle side of the stomach without defined margins (Figure 2). Esophagogastroduodenoscopy showed only mild gastritis.

**Figure 3:**
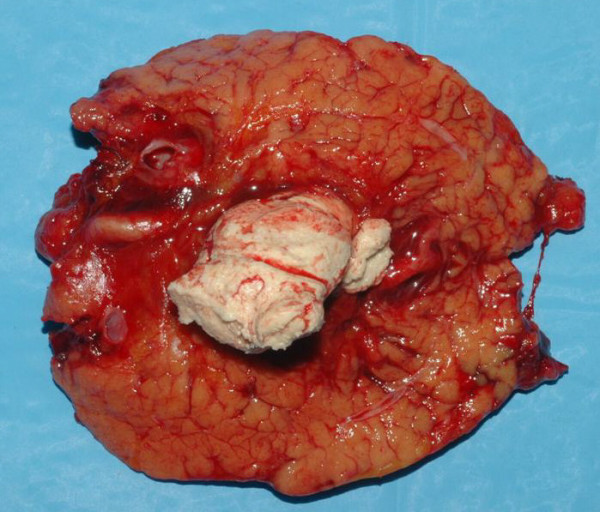
**Macroscopic view of the cut surface of the tumour**. Surgical specimen shows that the cyst is filled with finely granular, greyish white, keratinaceous and sebaceous material.

Considering the size of the lesion, we decided for resection without a preoperative biopsy and a cystic tumor, originated from the pancreatic tail was noted, therefore left pancreatectomy was achieved. At macroscopical evaluation the cyst, measured 8.5 × 3.0 cm, appeared encapsulated by a cystic wall and containing a greyish white material with "caseous" appearance characteristics of keratinaceous and sebaceous secretions (Figure 3).

**Figure 4:**
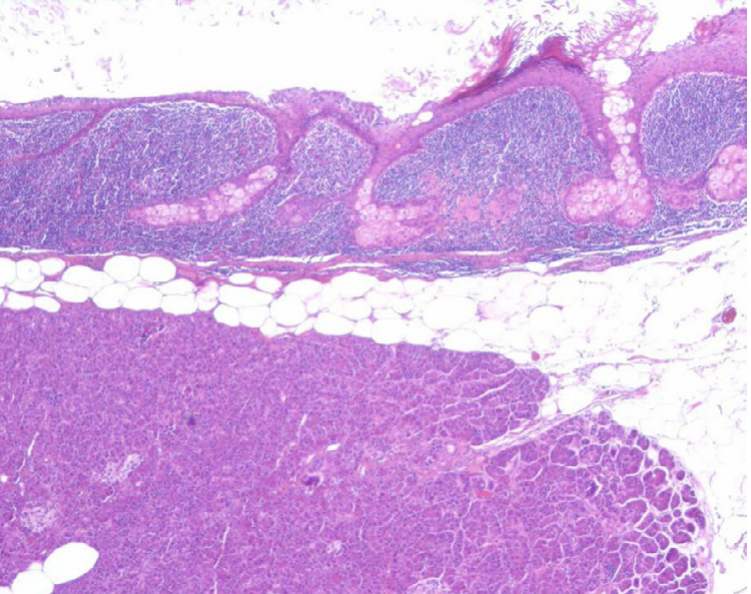
**Pathological findings of the cystic lesion**. Hematoxylin and eosin stain original magnification 40×. The dermoid cyst appears lined by keratinized squamous epithelium with sebaceous glands and immediately adjacent, dense sub epithelial lymphoid tissue that contains lymphoid follicles and germinal centres. Residual pancreatic tissue in the lower field.

**Figure 5:**
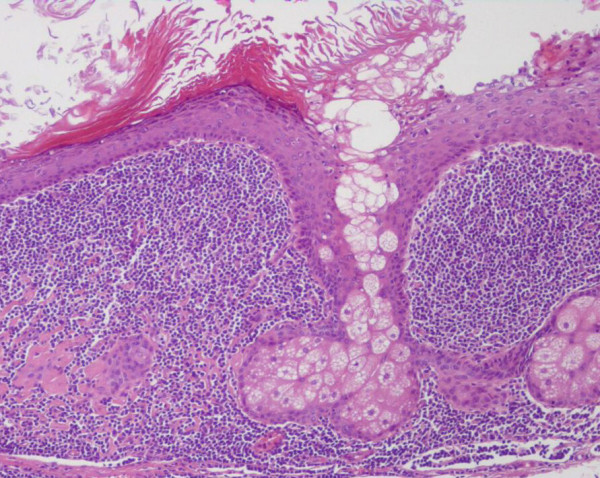
**Pathological findings of the cystic lesion**. Hematoxylin and eosin stain original magnification 100×. Note the keratinized squamous epithelium lining the cyst wall, the sebaceous gland in the underlying stroma and the dense lymphocytic infiltrate.

Histological evaluation revealed a benign teratoma characterized by an intense lymphoid reaction; the lymphoid tissue surrounding the mass contained few germinal centres lymphoid follicles, as for chronic flogistic reaction (Figure 4 and 5).

## Discussion

Pancreatic cystic lesions comprise a pathologically heterogeneous group of lesions that can be classified as congenital, inflammatory, and neoplastic and share many common clinical features. Identification of the different cystic lesions is still inadequate. There is ample debate regarding the diagnostic approach in such patients. Some observers consider the diagnosis intraoperative, whereas others choose to determine the need for surgery based on the results of biochemical and tumor markers in the cystic fluid obtained by CT or EUS-guided FNA.

Differential diagnosis of pancreatic dermoid cysts from the other two types of pancreatic cysts lined by squamous epithelium, e.g. lymphoepitelial cysts and epidermoid cyst, may be difficult preoperatively.

Dermoid cysts usually occur in a younger age group, and in contrast with lymphoepitelial cysts, which are more common in men, there is no gender predominance. Furthermore, mucinous epithelium, respiratory-type mucosa, sebaceous units and hair follicles are more readily identifiable in dermoid cysts rather than in lymphoepitelial or epidermoid cysts. Finally suppurative infections are more frequent in dermoid cysts rather than in other "squamous-lined" pancreatic cysts [24].

Given the absence of any known malignant potential of the above mentioned lesions, differential diagnosis between dermoid cyst and primary neoplastic cysts of the pancreas (e.g. mucinous cystic neoplasms, intraductal papillary mucinous neoplasms and intraductal oncocytic papillary neoplasms) appears more clinically significant.

The traditional serum markers, such as CEA and CA19-9, would be expected to be significantly lower in dermoid cysts rather than in other pancreatic cystic neoplasms; however, our experience does not confirm this prediction [25].

It was suggested that the combined use of ultrasonography, enhanced computed tomography and magnetic resonance cholangiopancreatography might allow differentiation from cystic lesions such as mucinous cystic tumors and intraductal papillary-mucinous tumors. Since cystic teratomas enclose keratinous and sebaceous material, they are echogenic, appearing as solid masses on US. Enhanced CT shows their cystic nature, with values slightly higher than water, and MRCP reveals defects of internal signals [20].

Moreover, the differential diagnosis of dermoid cysts from the rare cystic forms of solid pancreatic tumors (solid pseudopapillary tumors, ductal adenocarcinomas, cystic islet cell tumors, and acinar cell cystadenocarcinoma) is a clinical challenge [26].

Imaging may be not specific and the radiological appearance of these lesions differs according to the proportions of the various tissues enclosed in the cysts. CT accurately estimates the density of all the included tissues, such as soft tissue, fluid, fat, calcification, and teeth. MRI can also be performed for further characterization and study of anatomic relations [14,20,21].

Recently, EUS has been shown to have a high sensitivity to detect pancreatic masses. EUS-guided fine needle aspiration (FNA) cyto-histolology has been shown to be a safe, reliable and cost-effective tool to detect pancreatic masses, including malignant or benign neoplasms, pseudo cysts, and reactive changes. Pancreatic EUS-guided FNA cyto-histolology, coupled with the possibility of biochemical and tumor marker measurement in the cystic fluid, seems to allow an accurate and safe diagnosis without the risk, the cost, and the time expenditure of an open biopsy or laparotomy. A recent review about image-guided FNA biopsy of the pancreas revealed a relatively high overall sensitivity (64%–98%), specificity (80%–100%), and positive predictive value (98.4%–100%) [27].

Nevertheless, preoperative diagnosis of cystic lesions of the pancreas remains difficult, especially in presence of larger lesions (>3 cm), as suggested by Volmar [28] who reported, in a review of 1000 cases of US, TC or EUS-guided FNAC of pancreas, a false positive rate of 0.3% and a false negative rate of even 14.3% when compared with histology and clinical follow-up. In addition, the quality and the proper handling of the aspirated sample are crucial to the success of the diagnosis and the performance of biochemical and tumoral markers appears of limited help in the differential diagnosis of cystic lesions of the pancreas [28].

Further, the risk of cancer seeding along the needle tract during percutaneous sampling of pancreatic masses is noteworthy; the risk of peritoneal carcinomatosis appears to be lower with EUS-guided FNA compared with transcutaneous sampling methods [29].

Treatment of dermoid cysts, reported in previous experiences, consists of surgical removal; conservative treatment has not been described and, in most cases, cystectomy was performed [1-21]. Several factors interplay in the treatment choice: diagnostic accuracy of preoperative diagnostic tools, clinical presentation and type of symptoms, cystic neoplasm site, type and safety of resection.

It was suggested that conservative procedure, such as preservation of the pylorus in proximal pancreatectomy, segmental resection for isthmic/body tumors, and preservation of the spleen in distal pancreatectomy, is suitable to limit late sequelae. Simple cystic enucleation seems suitable for peripheral lesions, but very high rates of mortality and morbidity were reported in previous series [30]. In a previous revision of the results of surgery for pancreatic serous cystadenoma, Pyke et al[30] described complications that required reoperation in four of eight patients who underwent enucleation of the tumor without formal anatomic pancreatectomy. The routine use of prophylactic octreotide might be considered to decrease the high rate of pancreatic fistula. The decision to perform pancreatic resection is easier, as in our case, for body/tail tumors, which are not subject to surgical mortality, than for proximal cystic lesions, which have an operative mortality rate of up to 2% [31].

## Conclusion

Given the benign nature of the dermoid cyst and considering the improvement in preoperative imaging together with the availability of FNA, that seems a promising tool for preoperative evaluation of pancreatic cystic lesions, it was suggested that if apt diagnoses were made, teratomas would not needed to be resected [2]. Nevertheless, until more cases of dermoid cyst will be identified to further elucidate its natural history and improve the reliability of preoperative diagnosis, surgical resection should still be considered the standard therapy, in suitable patients, to exclude malignancy.

## Competing interests

The author(s) declare that they have no competing interests.

## Authors' contributions

**GT**: critical review

**MGM**: manuscript preparation and critical review

**CN**: critical review

**FC**: literature review and manuscript preparation

**DA**: data collection and literature review

**FS**: manuscript preparation

**AMF**: critical review
